# Tradeoffs in the Evolution of Caste and Body Size in the Hyperdiverse Ant Genus *Pheidole*

**DOI:** 10.1371/journal.pone.0048202

**Published:** 2012-10-25

**Authors:** Terrence P. McGlynn, Sarah E. Diamond, Robert R. Dunn

**Affiliations:** 1 Department of Biology, California State University Dominguez Hills, Carson, California, United States of America; 2 Department of Biology, North Carolina State University, Raleigh, North Carolina, United States of America; Arizona State University, United States of America

## Abstract

The efficient investment of resources is often the route to ecological success, and the adaptability of resource investment may play a critical role in promoting biodiversity. The ants of the “hyperdiverse” genus *Pheidole* produce two discrete sterile castes, soldiers and minor workers. Within *Pheidole*, there is tremendous interspecific variation in proportion of soldiers. The causes and correlates of caste ratio variation among species of *Pheidole* remain enigmatic. Here we test whether a body size threshold model accounts for interspecific variation in caste ratio in *Pheidole*, such that species with larger body sizes produce relatively fewer soldiers within their colonies. We evaluated the caste ratio of 26 species of *Pheidole* and found that the body size of workers accounts for interspecific variation in the production of soldiers as we predicted. Twelve species sampled from one forest in Costa Rica yielded the same relationship as found in previously published data from many localities. We conclude that production of soldiers in the most species-rich group of ants is regulated by a body size threshold mechanism, and that the great variation in body size and caste ratio in *Pheidole* plays a role in niche divergence in this rapidly evolving taxon.

## Introduction

Organisms have limited resources to invest, yet demonstrate great variability in how those resources are invested [1]–[4]. How do we explain this remarkable variation in life history? Societies provide a unique opportunity to explore this question in that they can vary through the differential allocation of work among specialized individuals, in contrast to allocation within individuals. Societies vary from those in which most individuals perform similar roles, to those in which thousands of different jobs are undertaken [5]. In insect societies, colonies produce morphologically distinct castes that differ in their function for the colony. Sterile workers, which are morphologically distinct from reproductive individuals, are typically produced in greater quantity than reproductives. In some insect societies, two discrete sterile castes are produced, which differ in body size, morphology and behavioral role within the colony. When there are two discrete sterile castes, the larger caste members (which often perform a defensive function) are often called “soldiers.” For clarity, we consistently refer to the smaller worker caste as “minor workers.”

The investment into a soldier caste allows species with similar morphologies to inhabit distinct niches by altering the relative investment into soldiers. More soldiers can allow a colony to better defend a food item, whereas more workers can allow it to better find food in the first place [6]. Wilson [7] described such castes, and the varied life histories they allow, as the pinnacle of optimization. The evolution of caste ratios in response to variation in ecological conditions may provide at least a partial explanation for the evolutionary success and ecological dominance of insect societies [8]. In ants, the mechanisms underlying evolutionary changes in the ratios of soldiers and workers have long and often been speculated upon [9], but are not well known.

There is growing evidence to suggest predictable associations between body size and caste differentiation within some species of ants. In ants of the genus *Pheidole*, all nascent worker larvae have the potential to become minor workers or soldiers. Wheeler and Nijhout [10] described the presence of a developmental threshold in *Pheidole*. In this well-supported model, larvae surpassing a threshold body size in their last instar will become soldiers, whereas larvae below this size will become minor workers. Thus, the proportion of soldiers produced in a colony is predicted by the distribution of larval body sizes in a colony.

Soldier ratios vary greatly in natural populations, among species and among populations within a single species. Yang et al. [11] sought to understand how body size influenced the proportion of soldiers produced by three distinct populations of *Pheidole morrisi*, that occurred in particularly different environmental conditions. The percent of majors significantly differed among sites, ranging from 10% to 15%. This difference in soldier proportion was tightly coupled with differences in the body sizes of adult minor workers and soldiers in each population. Within *P. morrisi*, the shifts in body size that tracked environmental variation were associated with changes in soldier production. Yang e al. [11] proposed two models to account for shifts in soldier proportion. In the first model, the threshold for soldier determination would vary, which would be consistent with a negative association between the proportion of soldiers and the body sizes of minor workers and soldiers. In the second model, the threshold for soldier determination would be fixed, and the distribution of larval sizes would increase, thus producing a higher proportion of soldiers. They found that decreases in the body size of adult workers and soldiers were accompanied by an increase in the proportion of the soldiers produced in the colonies. This result was consistent with the first, “threshold shift,” model, in which the threshold body size for soldier determination shifted with the body size distribution in the population. To date, the relationship between body size and soldier ratio described by Yang et al. has only been evaluated among populations within the same species. If this pattern is more widely distributed across the genus *Pheidole*, then interspecific differences in soldier production may be explained by lowering the body size threshold. The hyperdiverse genus of *Pheidole* does not demonstrate tremendous diversification in terms of morphology, but does contain great variation in both body size and caste ratio [12]. To understand the causes and consequences of the evolution of species richness in *Pheidole*, we must understand the ways in which body size and caste evolve and are associated with one another.

Here we use a comparative approach to examine interspecific variation in the body sizes and caste ratios of minor and soldier workers 26 *Pheidole* species. We expect Yang's observed pattern within the populations of *Pheidole morrisi* to be recapitulated among species, such that species with smaller body sizes produce a greater fraction of soldiers within their colonies.

## Materials and Methods

**Figure 1:**
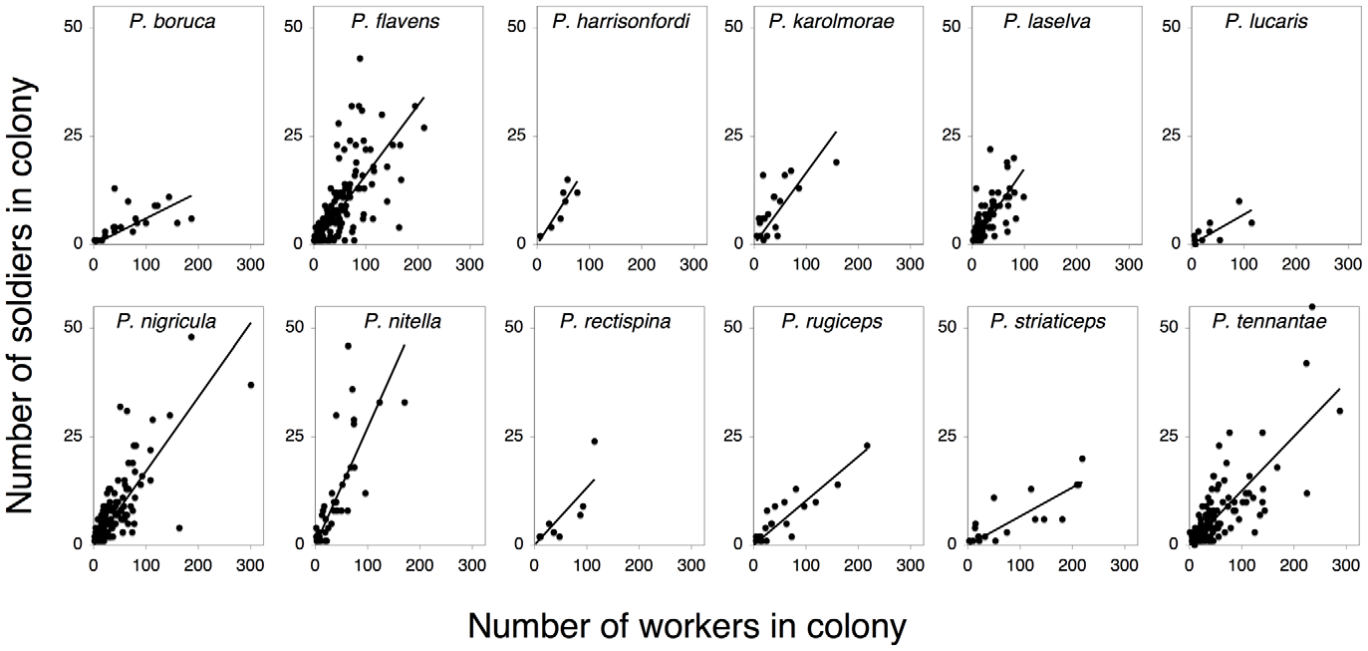
The relative production of soldiers in twelve species of *Pheidole*, collected from La Selva Biological Station, Costa Rica.

Because most species of *Pheidole* are tropical and relatively few tropical ant species have been studied in detail, a challenge in considering the evolution of caste is documenting the variation in caste and body size in the first place. Our first approach was to study body size and proportional investment in soldiers at a single well-studied field site in lowland tropical rainforest in northeastern Costa Rica within La Selva Biological Station (Supplementary Information), where at least 700 person-days of sampling approximately 10^6^ twigs in total allowed us sufficient sample sizes of 12 species of the genus *Pheidole*. (Permits for research and collecting were obtained through the Costa Rican Governmental Ministry of Energy, Environment and Telecommunication.) Whole colonies of *Pheidole* spp. were collected using an exhaustive “intensive sampling” protocol [13] during two sampling events in 1997 and 2004, the latter in the CARBONO plot network, a series of sites throughout La Selva established for collaborative research on rainforest processes. Colonies were identified to species using the Ants of Costa Rica (http://ants.biology.utah.edu/~longino/AntsofCostaRica.html), and the number of individuals comprising the soldier and minor worker castes were counted. All of the species identified were verified by referencing voucher specimens that are currently deposited at the Costa Rican National Institute of Biodiversity (INBio). The ordinary least squares slope of soldier number as a function of total worker number (soldiers plus minors), with the intercept constrained to zero, was used to estimate proportional investment in soldiers for each of the 12 species (Fig. 1). To complement these data, proportional investment in soldiers for 16 *Pheidole* species was obtained from the primary literature (Table S1) [10], [11], [14]–[17]. Two of these species were shared with the Costa Rica species, yielding 26 unique species. All data from Costa Rica and the primary literature were obtained from comparable, unmanipulated, colonies. While data on the caste ratios of other species of *Pheidole* may exist, the twenty six species considered here include all of those species that resulted in thorough searches of Web of Science and JSTOR (both accessed May 2011) using the terms “caste” and “*Pheidole*.”

Although our response variable is the proportional investment in soldiers–and proportional data are often most appropriately modeled using a binomial distribution (logistic regression)–we model these data using a Gaussian distribution for several reasons: 1) the Shapiro-Wilk test for normality indicated the distribution of proportional investment in soldiers was not significantly different from a normal distribution (W = 0.951, p = 0.189), 2) plots of the fitted values versus residuals for models of proportional investment in soldiers as functions of minor and major worker head width (including those corrected for phylogeny) exhibited no clear structure, 3) interpretations of the models are simpler using the data scale rather than the transformed logit scale, and 4) models based on logit-transformed proportional investment in soldiers yielded qualitatively similar results as the untransformed data, with soldier investment significantly negatively associated with both minor and major worker head width.

**Figure 2:**
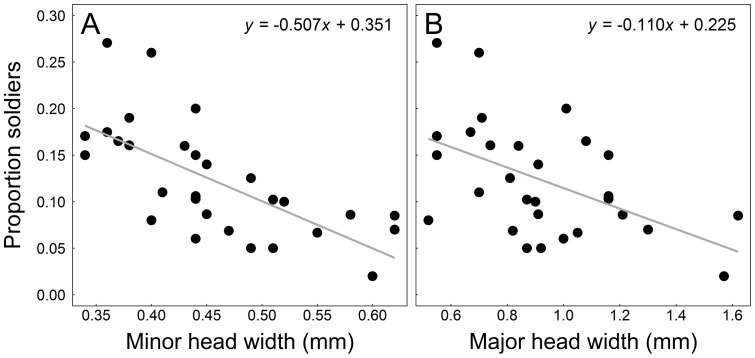
Proportional investment in soldiers is a function of body size. Panel A indicates the relationship between soldier investment and minor worker head width (mm); Panel B indicates the relationship between soldier investment and soldier head width (mm). Linear regressions are indicated with grey solid lines.

Mean ant body size was assessed for soldier and minor castes of each species. Ant head width, which scales isometrically with other aspects of ant body size [18], was used as a proxy of overall body size. The head widths of mounted field-collected ants were digitized (using full-faced images captured to scale) and measured with ImageJ 1.43u (Wayne Rasband, NIH, USA).

We examined the relationship between soldier investment and ant body size using linear regressions, in which proportional investment in soldiers was considered a continuous response, and minor head width was considered a continuous fixed effect. Substantial collinearity of minor head width and soldier head width (see Results) prevented inclusion of soldier head width in this model; therefore, we performed a separate regression where soldier head width was considered a continuous fixed effect.

To examine the potential influence of phylogenetic autocorrelation, we performed a phylogenetic generalized least squares (PGLS) model [19] of the relationship between soldier investment and ant body size, using the 11 *Pheidole* species represented in both the caste ratio and body size trait dataset considered here and in the *Pheidole* phylogeny [20], with branch lengths. Mean trait values were calculated for *P. morrisi*, the only species with multiple species-level replicates (n = 3) on the phylogeny. We fit PGLS models where the degree of phylogenetic autocorrelation (Pagel's λ) [21] was simultaneously co-estimated. Lambda is a measure of phylogenetic inertia, or how closely the structure in the model residuals resembles the structure of the phylogeny; values range from 0 to 1, with greater values indicating greater phylogenetic structure. All statistical analyses were performed using R (version 2.11.1; R Development Core Team).

## Results

We found considerable interspecific variation in soldier ratio among the 26 *Pheidole* species: proportional investment in soldiers varied by an order of magnitude, ranging from 0.02 to 0.27. Importantly, the variation in soldier investment was significantly related to variation in ant body size. Greater investment in soldiers was associated with smaller ant body size (in both minors: β = −0.52± SE  = 0.11, *t* = −5.0, df  = 28, *p*<0.0001, and soldiers: β = −0.11± SE  = 0.036, *t* = −3.1, df  = 27, *p* = 0.0049; Fig. 2), as predicted by the developmental threshold model. Indeed, ant body size alone explained 47 and 26% of the variation in soldier investment, based on minor worker and soldier worker body size, respectively. Soldier and minor body sizes were, in turn, strongly positively correlated (*r* = 0.78; *t* = 6.5, df  = 27, *p*<0.0001).

To account for the potential influence of methodological-based differences among the Costa Rica contribution and the primary literature contribution to the dataset, we re-performed analyses of soldier investment using only the Costa Rica data, for which we were certain methodologies were comparable. These results were qualitatively similar to the results from the complete dataset (soldier investment as a function of ant body size for minors: β = −0.70± SE  = 0.18, *t* = −3.8, df  = 10, *p* = 0.0035, *r* ^2^ = 0.59, and soldiers: β = −0.25± SE  = 0.080, *t* = −3.1, df  = 10, *p* = 0.011, *r* ^2^ = 0.50).

**Figure 3:**
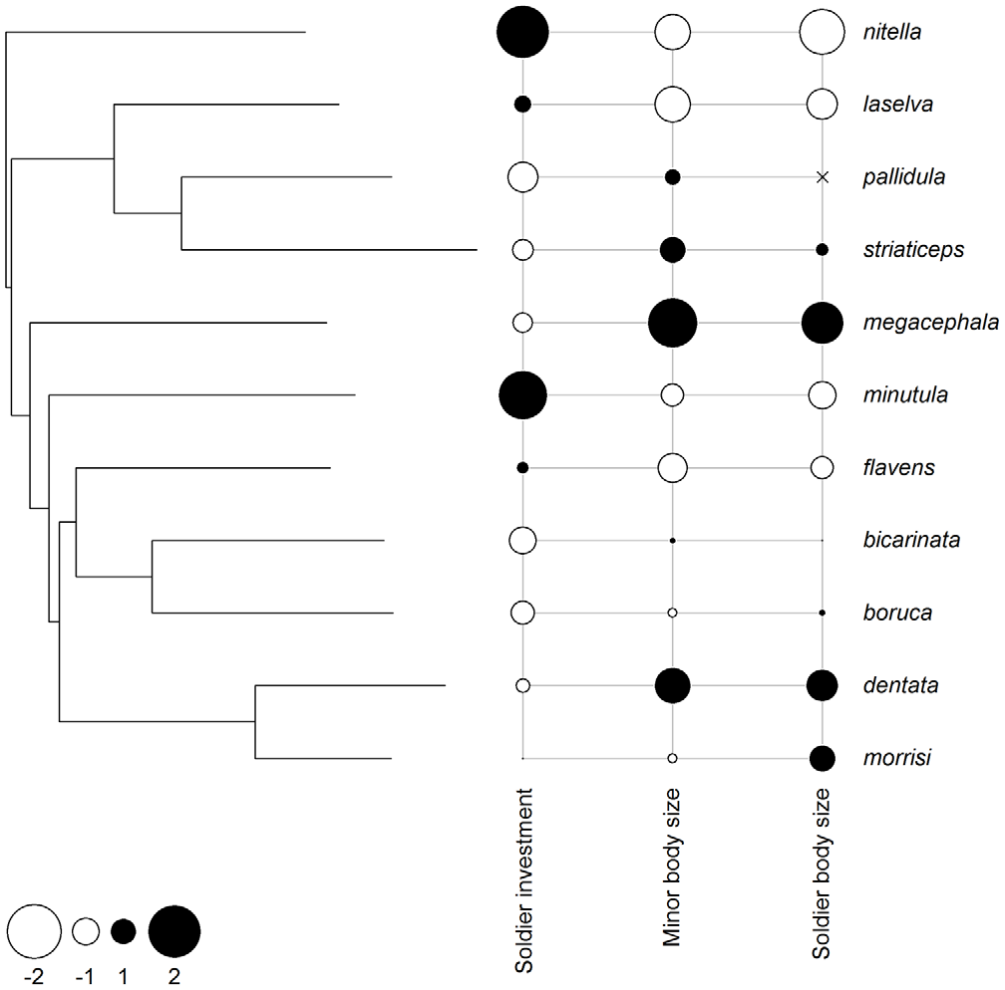
Proportional investment in soldiers, minor worker body size and soldier body size mapped on to the *Pheidole* phylogeny. Traits are scaled and centered, with filled circles representing values above the mean trait value and open circles representing values below the mean trait value. Larger circles indicate greater distance from the mean trait value. “×” indicates trait data not available. Mean trait values are reported when data from multiple populations of a single species were available.

Although phylogenetic autocorrelation was relatively high in our PGLS models of soldier investment as a function of ant body size (λ = 0.99 in models based on either worker body size or soldier body size, a value consistent with Brownian trait evolution; Fig. 3), our major result was qualitatively unaltered: greater proportional investment in soldiers was associated with smaller ant body size (minors: β = −0.61± SE  = 0.19, *t* = −3.3, *p* = 0.009, df  = 9, *r* ^2^ = 0.55; soldiers: β = −0.26± SE  = 0.065, *t* = −4.1, *p* = 0.0037, df  = 8, *r* ^2^ = 0.67). Estimates of phylogenetic signal in the traits themselves, based on Blomberg's K [22], including soldier investment (K = 1.0), and soldier and worker body size (K = 1.0, and K = 0.83, respectively) were at or near the expected value of 1 under Brownian trait evolution (though in each case, the variance of phylogenetically independent contrasts was not significantly different from a null model of shuffling trait values across the tips of the phylogeny, likely owing to the small number of samples in this analysis).

## Discussion

Among the most interesting aspects of societies is the extent to which they differ in how they invest their resources, particularly with regard to the relative investment in different tasks or castes. Differences among species in caste ratios have long been implicated as being key to the diversification of social organisms and their niches [7]. Here we used a comparative approach to test the hypothesis that caste ratios evolve among species of the hyperdiverse genus *Pheidole* via the evolution of body size and a corresponding shift in the body size threshold for soldier determination. We found evidence for a tradeoff between species' proportional investment in the soldier caste and body size. Our results suggest an important role for life history tradeoffs and developmental architecture [23] in shaping the evolution of caste ratios.

Developmental threshold models of body size have been found to underlie many non-genetically based polymorphisms [11], [24]–[27]. We found that the threshold model underlying proportional investment in soldiers across populations of *Pheidole morrisi* [11] also appeared to explain soldier investment across several *Pheidole* species. As predicted by the threshold model, we found a negative association between species' investment in soldiers and worker body size, and a positive relationship between minor and soldier worker size (Fig. 2). The former component suggests greater investment in soldiers is achieved by a lowering of the size threshold at which undifferentiated workers become soldiers, rather than an increase in the larval distribution for body size.

Variation in soldier investment influences a colony's fitness: optimal (and potentially unrealized) investment in soldiers is a function of body size and body size thresholds in addition to other factors [28], [29]. For example, changes to the competitive or resource environments have modestly influenced the rate of soldier production, in two species in the present study [29], [30], though the magnitude of such effects was well within the observed variation in caste ratio as a function of body size from our analyses here. It is conceivable that these changes were associated with changes in body size thresholds and body size, though it is unclear since neither of these studies included measurements of body sizes. Competitive environments might also influence soldier ratios over evolutionary time scales, though our results suggest that such changes in caste ratio would be associated with concomitant changes in body size, thereby limiting evolution along the orthogonal axis. It would be interesting to sample more *Pheidole* species to search for species with large body sizes and high proportions of soldiers, if such species exist, to understand how they break the observed pattern. The covariance between body size and caste ratio does not necessarily require a shared evolutionary history, as we found little phylogenetic evidence to suggest worker body size or caste ratio were strongly conserved, as K values were not >> 1 (Fig. 3). The more parsimonious conclusion is that these traits have coevolved. Because ancestral potentialities in caste determination in *Pheidole* may result in the independent evolution of novel castes following the same developmental pathways for supersoldiers [31], a fruitful avenue of investigation may be to test whether body size should inform the upregulation of the pathways triggering the development of soldiers.

We are careful to point out here that our results relate to a single genus of ants of which we have sampled a small subset of species. We suspect our results generalize to many species of *Pheidole*, in part because the species of *Pheidole* we considered included most of the major clades so far identified in the genus [20], though it is conceivable lineages of the genus *Pheidole* deviate from the threshold model for which we found support here. Such lineages, if they exist, deserve careful study and might already be known of by an ant biologist somewhere. Our results suggest that the study of caste ratios in body size in other ant genera with soldier castes would be very interesting.

## Supporting Information

Table S1 **Proportional investment in soldiers of** ***Pheidole*** **from the current study and previously published reports.**
(PDF)
