# Supplementary material for: Tradeoffs in the Evolution of Caste and Body Size in the Hyperdiverse Ant Genus Pheidole
Source: PLoS One. 2012 Oct 25;7(10):e48202. doi: 10.1371/journal.pone.0048202 (PMC3485035; doi:10.1371/journal.pone.0048202)
Supplement: Table S1 — Proportional investment in soldiers of Pheidole from the current study and previously published reports. (PDF) [file pone.0048202.s001.pdf]

Table S1. Proportional investment in soldiers of *Pheidole* from the current study and previously published reports. In studies that reported multiple values from the same locality, the mean soldier ratio of unmanipulated colonies is provided.

Species	Soldier investment	Source	Locality
P. bicarinata	0.050	Wheeler & Nijhout 1981	North Carolina, USA
P. boruca	0.060	Current study	Costa Rica
P. dentata	0.086	Wilson 1984	Florida, USA
P. distorta	0.020	Wilson 1984	Manaus, Brazil
P. embolopyx	0.20	Wilson 1984	Manaus, Brazil
P. fervida	0.10	Ito & Higashi 1990	Hokkaido, Japan
P. flavens	0.16	Current study	Costa Rica
P. guilelmimuelleri	0.085	Wilson 1984	Manaus, Brazil
P. hortensis	0.14	Wilson 1984	Sri Lanka
P. karolmorae	0.18	Current study	Costa Rica
P. laselva	0.069	Current study	Costa Rica
P. lucaris	0.17	Current study	Costa Rica
P. megacephala	0.070	Wilson 1984	Florida, USA
P. mendicula	0.080	Wilson 1984	Manaus, Brazil
P. minutula	0.26	Wilson 1984	Manaus, Brazil
P. morrisi (FL)	0.15	Yang et al. 2004	Florida, USA
P. morrisi (NC)	0.10	Yang et al. 2004	North Carolina, USA
P. morrisi (NY)	0.11	Yang et al. 2004	New York, USA
P. multispina	0.11	Kaspari & Byrne 1995	Costa Rica or Panama
P. nigricula	0.17	Current study	Costa Rica
P. nigricula	0.15	Kaspari & Byrne 1995	Costa Rica or Panama

Species	Soldier investment	Source	Locality
P. nitella	0.27	Current study	Costa Rica
P. pallidula	0.042	Passera 1974	France
P. rectispina	0.087	Current study	Costa Rica
P. ruida	0.19	Current study	Costa Rica
P. rugiceps	0.10	Current study	Costa Rica
P. rugiceps	0.050	Kaspari & Byrne 1995	Costa Rica and Panama
P. specularis	0.16	Kaspari & Byrne 1995	Costa Rica or Panama
P. striaticeps	0.067	Current study	Costa Rica
P. tennantae	0.13	Current study	Costa Rica
